# Editorial: Dental biomaterials—addressing modern challenges and shaping future procedures

**DOI:** 10.3389/fdmed.2025.1773801

**Published:** 2026-01-12

**Authors:** Widowati Siswomihardjo, Mutlu Özcan, Arief Cahyanto, Siti Sunarintyas, Yosi Kusuma Eriwati

**Affiliations:** 1 Department of Dental Biomaterials, Faculty of Dentistry, Gadjah Mada University, Yogyakarta, Indonesia; 2 Clinic of Masticatory Disorders and Dental Biomaterials, Center for Dental Medicine, University of Zurich, Zurich, Switzerland; 3 Department of Clinical Sciences, College of Dentistry, Ajman University, Ajman, United Arab Emirates; 4 Department of Dental Materials Science, Faculty of Dentistry, Universitas Indonesia, Jakarta, Indonesia

**Keywords:** adhesive dentistry, antibacterial strategy, bioactive materials, bulk-Fill, dental biomaterials, fiber-reinforced, MTA, resin cement dual-cure

Dental biomaterials are central to modern clinical dentistry, shaping how we restore, regenerate, and preserve oral tissues across the lifespan. However, contemporary practice places increasingly complex demands on materials: durable adhesion under challenging conditions, reliability in conservative designs, antimicrobial and bioactive functions, compatibility with digital workflows, and performance that remains stable in a wet, biofilm-rich environment. In parallel, the field is being pushed toward more efficient and minimally invasive procedures, making technique sensitivity and workflow constraints as critical as traditional mechanical metrics.

The Research Topic Dental Biomaterials: Addressing Modern Challenges and Shaping Future Procedures brings together five contributions that reflect this shift toward materials and interfaces engineered for clinical reality. The papers collectively highlight how biomechanics, curing strategies, multifunctional chemistry, reinforcement concepts, and bio-inspired modifications can inform the next generation of restorative and endodontic procedures.

Wang et al. used a computational biomechanics approach to evaluate stress distribution and failure risk in mandibular incisors restored with resin-bonded fixed partial dentures using multiple CAD/CAM materials and restoration designs. Their findings reinforce that restoration geometry and loading direction strongly influence stress concentration and predicted failure probability, emphasizing that conservative prosthodontic solutions should be designed with reliability in mind rather than selecting materials based on strength alone.

Li et al. addressed a frequent clinical limitation in adhesive dentistry: reduced or compromised light transmission during cementation. By comparing curing modes and tracking micro-tensile bond strength over time, the study underscores that dual-curing systems may still exhibit performance differences when polymerization relies more heavily on chemical cure. This contribution supports the need to align cement selection and curing protocol with clinical access, restoration thickness, and realistic light delivery.

Altankhishig et al. investigated a bioactive universal adhesive containing calcium salt monomers, reporting mineralization potential, anti-demineralization effects, and antibacterial activity. This work reflects an important direction for dental biomaterials: the evolution of adhesives from passive bonding agents to multifunctional systems designed to stabilize the tooth-restoration interface and reduce secondary caries risk.

Obeid et al. compared physical-mechanical properties of flowable fiber-reinforced resin composites and bulk-fill Giomer composites, highlighting trade-offs relevant to posterior restorations. Their data provide a practical basis for selecting materials according to reinforcement strategy, polymerization behavior, and expected functional demands, and they illustrate why clinical decision-making should integrate both mechanical endpoints and placement efficiency.

Finally, Shetty et al. explored a bio-inspired modification of mineral trioxide aggregate using sericin, aiming to improve biophysical behavior and antimicrobial efficacy. Such approaches are aligned with an emerging expectation for endodontic repair and pulp-preservation materials: optimized handling and sealing should be paired with biological and antimicrobial performance to support more predictable outcomes in compromised environments.

Taken together, the contributions in this Research Topic highlight several converging priorities for the field: (i) reliability-aware design and evaluation for conservative restorations, (ii) curing and polymerization strategies that remain robust under clinical constraints, (iii) multifunctional chemistries that target interfacial stability and biofilm-related challenges, and (iv) translational evidence that links laboratory performance to realistic use scenarios. We hope this collection supports clinicians navigating increasingly complex material choices and encourages researchers to design studies that better reflect the mechanical, biological, and workflow conditions that ultimately determine success in practice.

